# Nano-silver-decorated microfibrous eggshell membrane: processing, cytotoxicity assessment and optimization, antibacterial activity and wound healing

**DOI:** 10.1038/s41598-017-00594-x

**Published:** 2017-03-27

**Authors:** Menglong Liu, Gaoxing Luo, Yuzhen Wang, Rui Xu, Ying Wang, Weifeng He, Jianglin Tan, Malcolm Xing, Jun Wu

**Affiliations:** 1 Institute of Burn Research, State Key Laboratory of Trauma, Burn and Combined Injury, Southwest Hospital, the Third Military Medical University, Chongqing, 400038 China; 2 Chongqing Key Laboratory for Disease Proteomics, Chongqing, 400038 China; 3 Department of Mechanical Engineering, University of Manitoba, Winnipeg, MB R3T 2N2 Canada; 4 Department of Biochemistry and Medical Genetics, University of Manitoba, Winnipeg, MB R3T 2N2 Canada; 5 Children’s Hospital Research Institute of Manitoba, Winnipeg, Canada; 6 Department of Plastic and Burn Surgery, the First Affiliated Hospital of Shenzhen University, Shenzhen, 518035 China

## Abstract

An ideal wound dressing can both promote wound healing and prevent bacterial infection. Here, we report a potential dressing prepared by incorporating an optimized concentration of silver nanoparticles (AgNPs) into the microfibers of a natural eggshell membrane (EM) using environmentally friendly and mussel-inspired dopamine. Briefly, acid-treated EM was used as a porous membrane for polydopamine-reduced AgNPs synthesis. To obtain the optimal cytocompatible silver concentration, cellular attachment and MTT assay were performed with different concentrations of AgNPs. The morphology of the EM and AgNPs was confirmed by scanning electronic microscopy, scanning transmission electronic microscopy and Fourier transform infrared spectroscopy. The synthesized EM/AgNPs exhibited steady and safe AgNPs release, which was further tested for antibacterial activity against *Escherichia coli* and *Staphylococcus aureus* by disc diffusion method and bacterial suspension assay. Finally, in a murine full-thickness skin wound model, we found that EM/AgNPs could promote re-epithelialization, granulation tissue formation and wound healing via enhancing cell proliferation, as demonstrated by the expression of proliferating cell nuclear antigen (PCNA), and controlling inflammation response, as demonstrated by the expression of interleukin-1β (IL-1β). These findings suggest that EM/AgNPs may have a promising application in wound management.

## Introduction

Cutaneous wounds caused by burns, trauma, or other conditions, such as diabetic foot ulcers, can lead to serious infections, body fluid loss, as well as major medical burdens. Therefore, suitable wound dressings are necessary to be applied as a skin barrier during the wound healing process^1–4^. To meet this demand, an ideal wound dressing should exhibit good biocompatibility and appropriate porosity, as well as effective antibacterial activity, to accelerate wound healing^5, 6^. However, few clinical products can meet all these needs due to their mono-functionality, relatively complicated preparation procedures, and high cost; thus, developing such an ideal wound dressing for tissue regeneration remains a challenge^5, 7, 8^.

Infection is a deadly opponent to wound healing. Among all antibacterial components, silver nanoparticles (AgNPs) have been well demonstrated to effectively prevent bacterial infections in wound by damaging the bacterial membrane and DNA^9–12^. On the other hand, the cytotoxicity of AgNPs is a major concern for extended applications^13, 14^. Recent studies reported that AgNPs cytotoxicity was dose dependent and occurred at high dosages^15–17^. Thus, while it is necessary to optimize the controlled release of AgNPs from a scaffold, few studies have been focused on integrating this feature in the context of the wound healing process.

Eggshell membrane (EM), mainly composed of collagens type I, V, and X^18, 19^, is a natural biomaterial that is biocompatible because of its similarity to native extracellular matrix. Additionally, EM is easily obtained at low cost^2, 20^. Principally, EM is a semi-permeable membrane comprising a fibrous network that separates the eggshell from the egg white, and thus exhibits low porosity. EM has been used for biotemplate, sorbent, biosensor and burn treatment applications^20–23^. Recently, EM has been well demonstrated to be benefit for wound healing. Fernando Guarderas *et al*. found that EM could promote wound closure in the early stages of wound healing^24^, Jun *et al*. reported that EM accelerated tympanic membrane healing in acute traumatic tympanic membrane perforation^25^, and Yang Chuang *et al*. considered that EM could provide wound protection and pain relief over split-thickness skin graft donor sites^26^. Furthermore, Durmus, E *et al*. showed that EM combined with eggshell had positive effect for the regeneration of cranial defects^27^, and Yehuda Zadik reported EM could promote healing of the full thickness traumatic lip laceration^28^. In addition, Long Chen *et al*. prepared a nanocomposite by blending soluble EM and polyurethane for the application of wound dressing^29^. Ohto-Fujita *et al*. found that immobilizing hydrolyzed EM on phosphorylcholine polymer could provide an extracellular matrix environment for human dermal fibroblasts adhesion and growth^30^. Collectively, EM is a promising biomaterial for preparation of wound dressings.

Marine mussels can tightly adhere on the solid surfaces in the sea by secreting proteins containing 3,4-dihydroxyphenyl-L-alanine (DOPA) and lysine^31, 32^. Inspired by mussel adhesiveness, Messersmith and his colleagues found that dopamine (DA) can undergo oxidative self-polymerization under alkaline conditions to form polydopamine (PD), enabling biomolecular or cellular attachment, and the catechol group in PD has the ability for chemical reduction^33, 34^. The mussel-inspired DA has super adhesive activity and can create a polymerized layer on various surfaces of material^31, 35^. In addition, PD exhibits good biocompatibility and low toxicity, which are beneficial for cell and tissue regeneration^7, 36^. Therefore, it is feasible to use DA to synthesize controlled concentrations of AgNPs on the surface of EM, although the cytotoxicity of treatment with different concentrations and under different conditions has not yet been studied.

In this report, we present a simple, environmentally friendly, and efficient method to incorporate an optimized dose of AgNPs into biocompatible EM microfibers using the adhesive and reductive properties of PD. The cytotoxicity and antibacterial activity *in vitro*, as well as the effect on wound healing *in vivo*, of the prepared EM/AgNPs nanocomposite were evaluated. We hypothesized that the optimized nanocomposite can promote wound healing and efficiently kill bacteria.

## Results

### Characterization of acid- and alkali-treated EM

**Figure 1:**
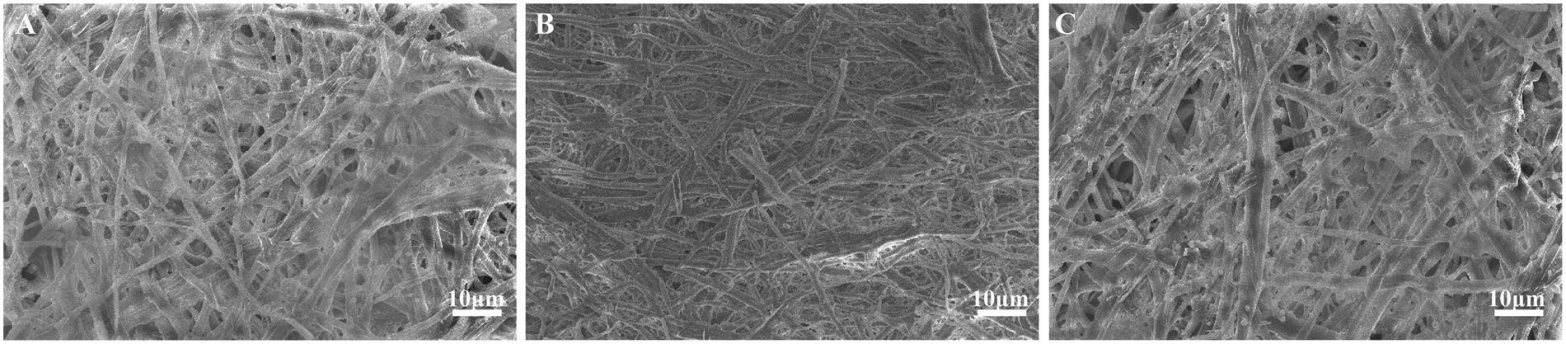
Characterization of acid- and alkali-treated EM. SEM images of EM treated for 72 hours with aqueous solutions of different pH values: (**A**) pH = 3; (**B**) DDH_2_O; and (**C**) pH = 11 (n = 3).

Since pristine EM has low porosity, we employed acid and alkali treatments to determine the ideal porosity. As shown in Fig. 1, all membranes presented structurally fibrous networks, and the diameter of interlacing protein fibers ranged from 1 μm to 3 μm, which was consistent with a previous review^2, 20^. However, by a scanning electronic microscopy (SEM) image analysis, the EM porosity increased from 42.1% to 46.1% and 45.0% after acid and alkali treatments, respectively, which are better for moisture and oxygen exchange. We also found that the acid-treated EM exhibited a better porous network via the removal of particulate impurities and the formation of a smoother surface compared with those that were untreated or treated with alkali aqueous solution. Therefore, we finally selected EM treated with a pH 3 solution for further use because of the higher porosity and improved porous network.

### Cytotoxicity of EM/AgNPs composite decorated with different AgNPs concentrations

**Figure 2:**
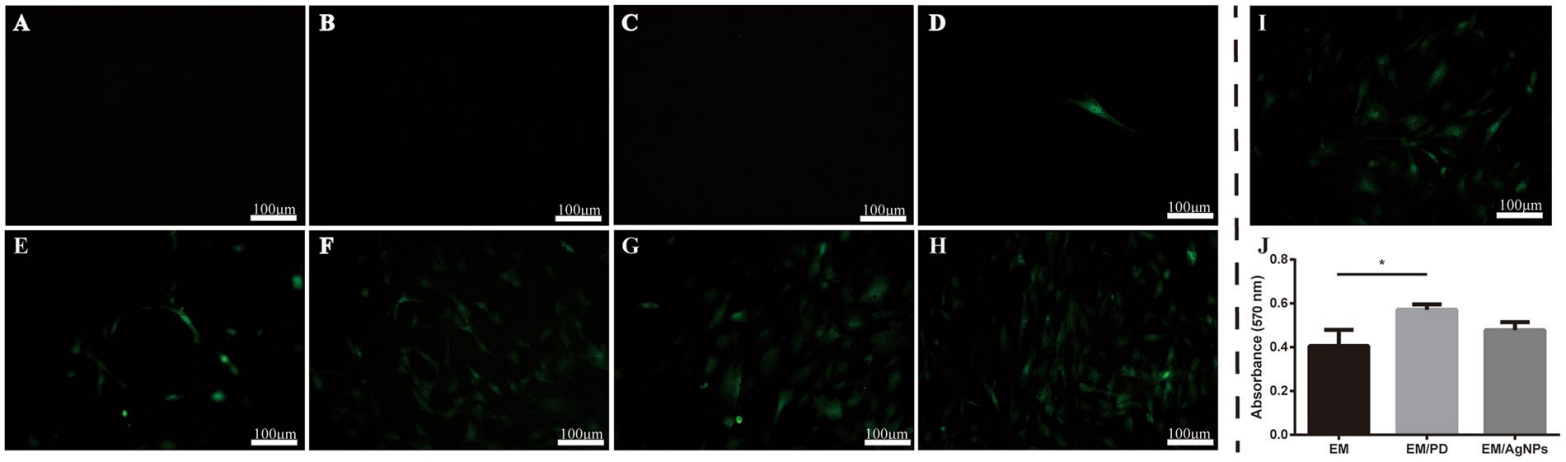
Cytotoxicity assessment of the optimized EM/AgNPs nanocomposite. Fluorescence microscopy images of GFP transgenic fibroblast attachment on the EM/PD films immersed in AgNO_3_ solutions of different concentrations: (**A**) 50 mM; (**B**) 10 mM; (**C**) 1 mM; (**D**) 100 μM; (**E**) 50 μM; (**F**) 30 μM; (**G**) 10 μM; and (**H**) 0 μM and (**I**) on EM. (**J**) Viability of fibroblasts cultured on EM, EM/PD and EM/AgNPs. The values are shown as the mean ± SD (n = 3).

To investigate the cytotoxicity of the EM/AgNPs, the composite films were immersed into silver nitrate (AgNO_3_) solutions of different concentrations, and then cell attachment was observed. As shown in Fig. 2A–C, no fibroblasts were attached at day 3 post-seeding on the membranes treated with a 50 mM, 10 mM or 1 mM AgNO_3_ solution for 18 h, indicating that the AgNPs formed *in situ* from these solutions were strongly cytotoxic. However, by decreasing the AgNO_3_ concentration, we found that fibroblasts could attach to the membranes treated with a 100 μM, 50 μM, 30 μM or 10 μM AgNO_3_ solution for 18 h (Fig. 2D–G), demonstrating the dose-dependent cytotoxicity of the AgNPs. In particular, membranes treated with a 30 μM or 10 μM AgNO_3_ solution showed cell attachment similar to that observed on EM/PD (Fig. 2F–H), suggesting good cytocompatibility. Considering the dose-dependent antibacterial activity of AgNPs^37^, we selected EM/PD treated with a 30 μM AgNO_3_ solution for 18 h for use in further research.

To further study the effect of EM/AgNPs on cell viability, an MTT assay was conducted. At day 3 post-seeding, the data showed no significant difference in cell viability between EM/PD and EM/AgNPs (Fig. 2J). In addition, EM/PD exhibited enhanced cell attachment and viability compared with EM (Fig. 2H–J).

### Characterization of the EM/AgNPs nanocomposite

**Figure 3:**
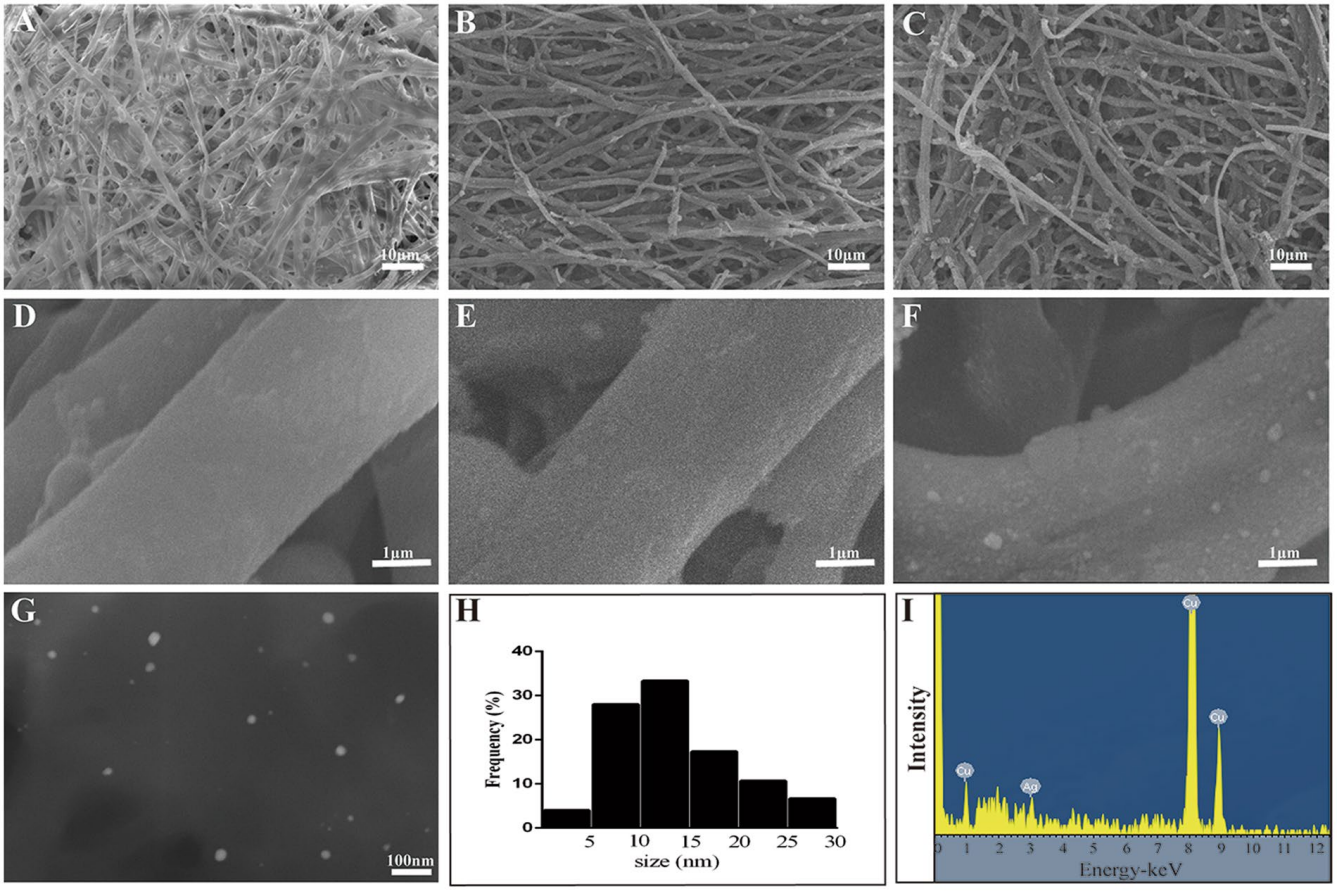
Characterization of the EM/AgNPs nanocomposite. SEM images of (**A**,**B**) EM, (**C**,**D**) EM/PD and (**E**,**F**) EM/AgNPs. (**G**) STEM images of AgNPs released from EM/AgNPs, (**H**) the size distribution of AgNPs, and (**I**) AgNPs EDS analysis result: the peak at 3 keV corresponds to Ag element, and the peaks at 1 keV, 8 keV and 9 keV correspond to Cu element (n = 3).

The morphology of the EM, EM/PD and EM/AgNPs was observed by SEM. As shown in Fig. 3A–C, there were no obvious differences among the EM, EM/PD and EM/AgNPs networks, and their porosity was 46.1%, 45.2% and 45.0%, respectively. The EM/PD and EM/AgNPs fibers were slightly rougher than the EM fibers, which might be attributable to the PD and AgNPs coating on the EM (Fig. 3D–F). AgNPs were observed to be scattered on the fibers of EM/AgNPs samples (Fig. 3F). The morphology and distribution of AgNPs were further investigated by scanning transmission electronic microscopy (STEM). As shown in Fig. 3G and H, AgNPs were typically spherical and uniformly distributed on the EM surface, and the average diameter of AgNPs was 13.9 nm, as determined by an Image J analysis. Together with STEM, the energy-dispersive X-ray spectroscopy (EDS) analysis of the EM/AgNPs nanocomposite demonstrated the presence of Ag (Fig. 3I).

**Figure 4:**
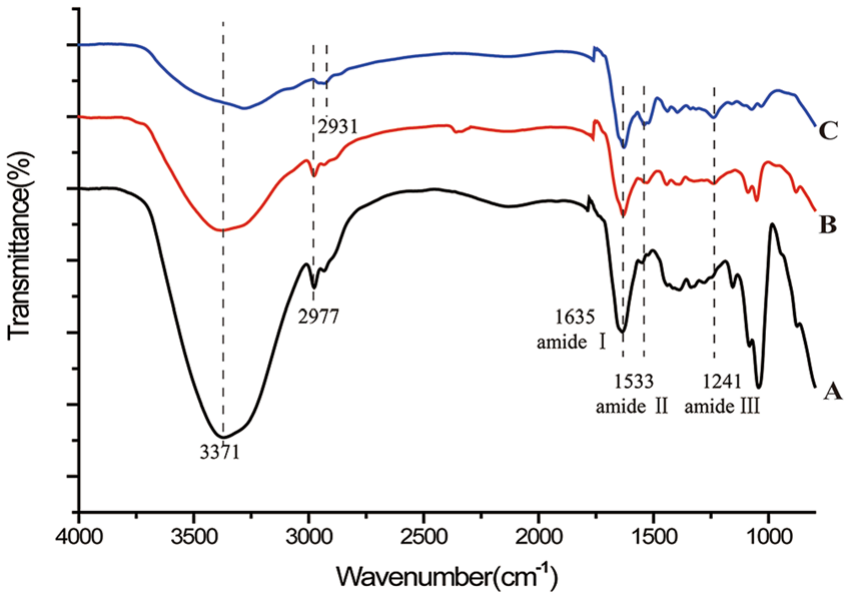
FTIR spectra of (**A**) EM, (**B**) EM/PD and (**C**) EM/AgNPs (n = 3).

The Fourier transform infrared (FTIR) spectroscopy (Fig. 4) analysis revealed that the spectrum of natural EM exhibited absorption bands at 3371 cm^−1^, corresponding to the stretching mode of O-H and N-H groups. The peaks at 1635, 1533, and 1241 cm^−1^ corresponded to the amide I (C=O stretching vibration), amide II (CN stretching/NH bending modes), and amide III bands (CN stretching/NH bending modes) of the glycoprotein mantle in EM, respectively^20^. The PD coating yielded a broad absorption band around 3300 cm^−1^ in the EM/PD and EM/AgNPs spectra, which may be attributed to the phenolic hydroxyl stretching vibrations of catechol groups^17^. With the deposition of AgNPs, intense absorption peaks at 1533 cm^−1^ and 1241 cm^−1^ were observed in the spectrum of EM/AgNPs, which might be due to the presence of AgNPs^6^. In addition, the absorption peak at 2977 cm^−1^ in the EM and EM/PD spectra shifted to 2931 cm^−1^ in the EM/AgNPs spectrum, suggesting the formation of AgNPs on the EM fibers.

### Silver ion release in PBS

**Figure 5:**
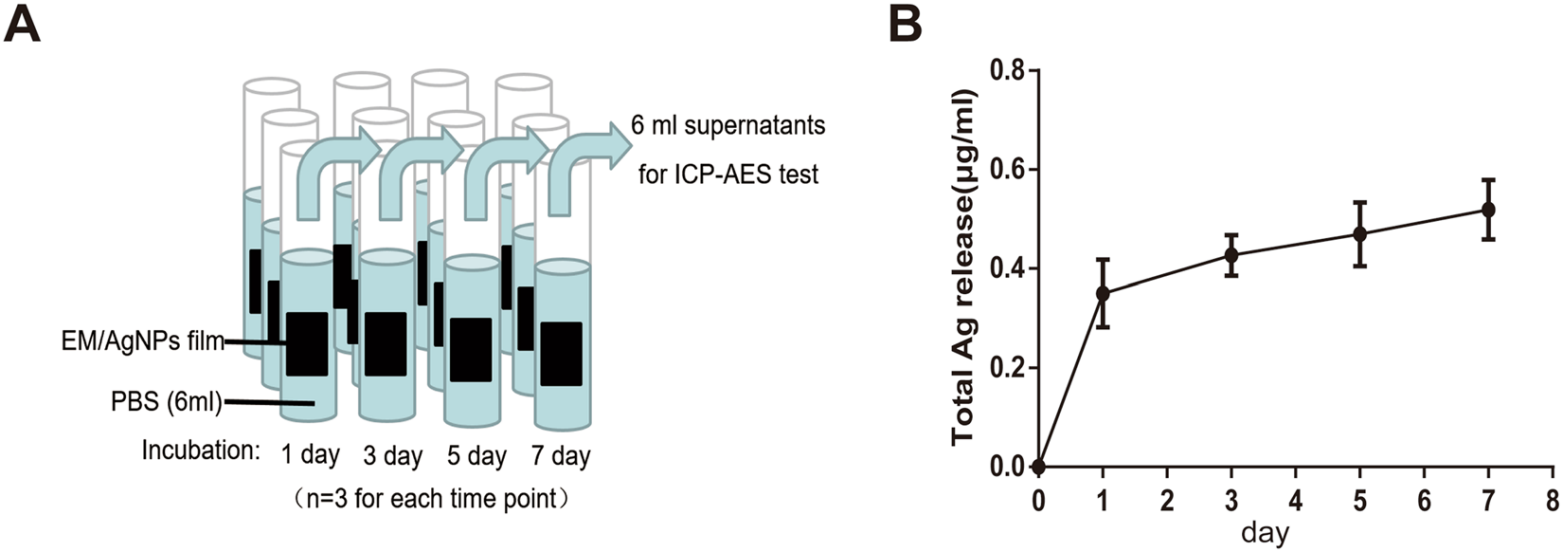
(**A**) Schematic diagram of ICP-AES assay. (**B**) Silver ion release in PBS from EM/AgNPs at days 1, 3, 5 and 7. The values are shown as the mean ± SD (n = 3).

The release profile of silver ions from EM/AgNPs was examined at days 1, 3, 5 and 7 in PBS using inductively coupled plasma-atomic emission spectrometry (ICP-AES) (Fig. 5). Silver ions were rapidly released from EM/AgNPs at day 1, which was subsequently followed by a relatively slow and sustained release. Briefly, the concentration of released silver ions was approximately 0.35 μg/mL at day 1. Furthermore, constant silver ion release could still be observed at day 7, suggesting that EM/AgNPs had a prolonged and steady antibacterial activity.

### Antibacterial assay

**Figure 6:**
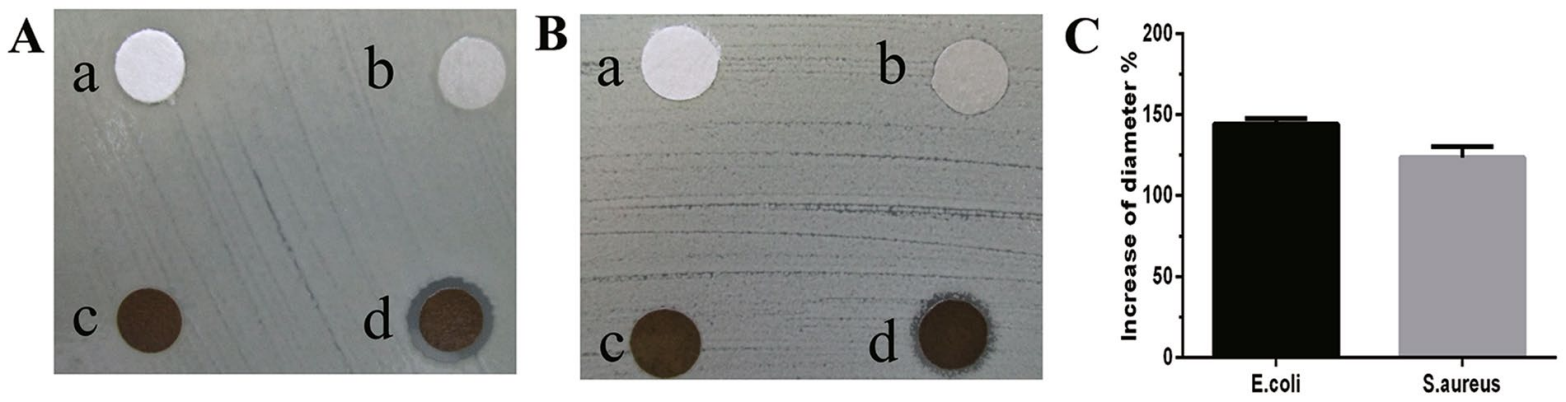
Evaluation of antibacterial activity against (**A**) *E. coli* and (**B**) *S. aureus* by the disc diffusion method using discs of (a) filter paper, (b) EM, (c) EM/PD and (d) EM/AgNPs. (**C**) Measurement of ZOI of EM/AgNPs against *E. coli* and *S. aureus*. The values are shown as the mean ± SD (n = 6).

A disc diffusion test was used to investigate the antibacterial activity of the EM/AgNPs. As shown in Fig. 6A,B, a clear inhibition zone was observed around EM/AgNPs films both for *E. coli* and *S. aureus*. However, inhibition zones were not found around discs of filter paper, EM or EM/PD films. The percentage of the increase in diameter for *E. coli* and *S. aureus* was 144.30% and 123.52%, respectively (Fig. 6C).

**Figure 7:**
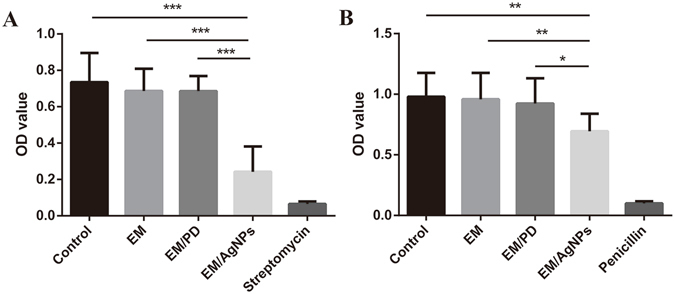
OD_600_ value measurements of (**A**) *E. coli* and (**B**) *S. aureus* bacterial suspensions after 24 h of incubation. The values are shown as the mean ± SD (n = 4).

To better understand the antibacterial activity of the EM/AgNPs, log-phase bacterial suspensions of *E. coli* and *S. aureus* were incubated together with different films, and the OD_600_ values were measured after 24 hours. The results showed that the OD_600_ value of the EM/AgNPs group was significantly lower than those of other groups, indicating that EM/AgNPs had a satisfactory antibacterial activity for both *E. coli* (Fig. 7A) and *S. aureus* (Fig. 7B). However, consistent with the results of the disc diffusion test, no significant differences were found between the control and either EM or EM/PD.

### Effect of the EM/AgNPs nanocomposite on wound healing *in vivo*

**Figure 8:**
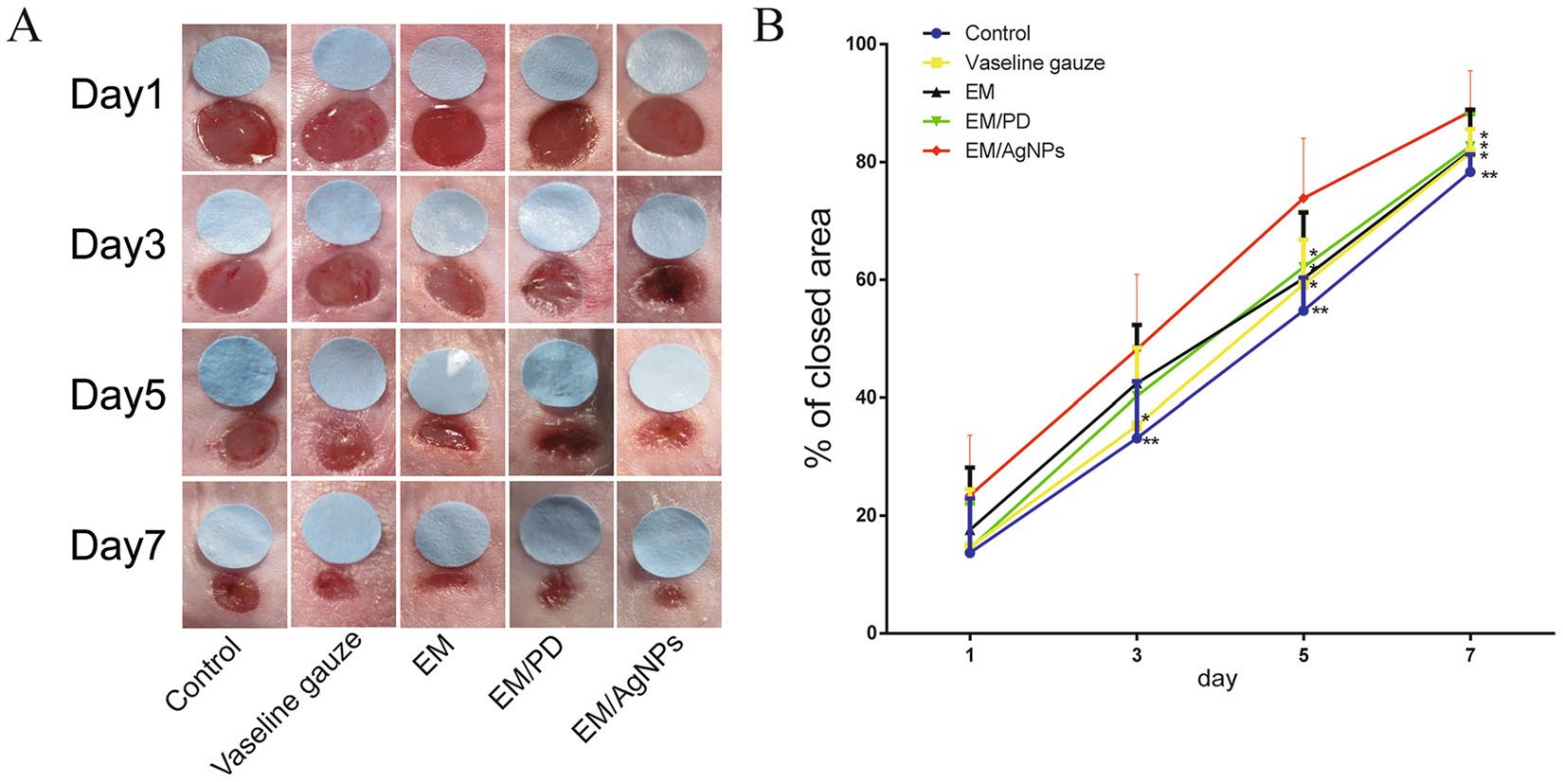
The effect of EM/AgNPs on wound healing. (**A**) The representative macroscopic appearance of wounds from the control, Vaseline gauze, EM, EM/PD and EM/AgNPs groups. (**B**) The area of wound closure at different time points. The values are shown as the mean ± SD (n = 7).

The *in vivo* wound healing efficacy of EM/AgNPs was investigated using a murine full-thickness skin defect wound model (Fig. 8). At day 1 post-surgery, the wound healing rate was similar in all groups. However, the area of wound closure in the EM/AgNPs group was significantly larger than that in the control and Vaseline gauze groups at day 3. Moreover, compared with all other groups, the EM/AgNPs group exhibited significantly accelerated wound healing at days 5 and 7. At day 7 post-surgery, approximately 88.6% of the wound area was closed, but the percentage of wound closure area was 78.3%, 81.6%, 81.9% and 82.7% in the control, Vaseline gauze, EM and EM/PD groups, respectively (Fig. 8B). Meanwhile, the skin around the wounds in the control, Vaseline gauze, EM and EM/PD groups was red and swollen for the first 5 days, indicating severe inflammation during the wound healing process (Fig. 8A).

**Figure 9:**
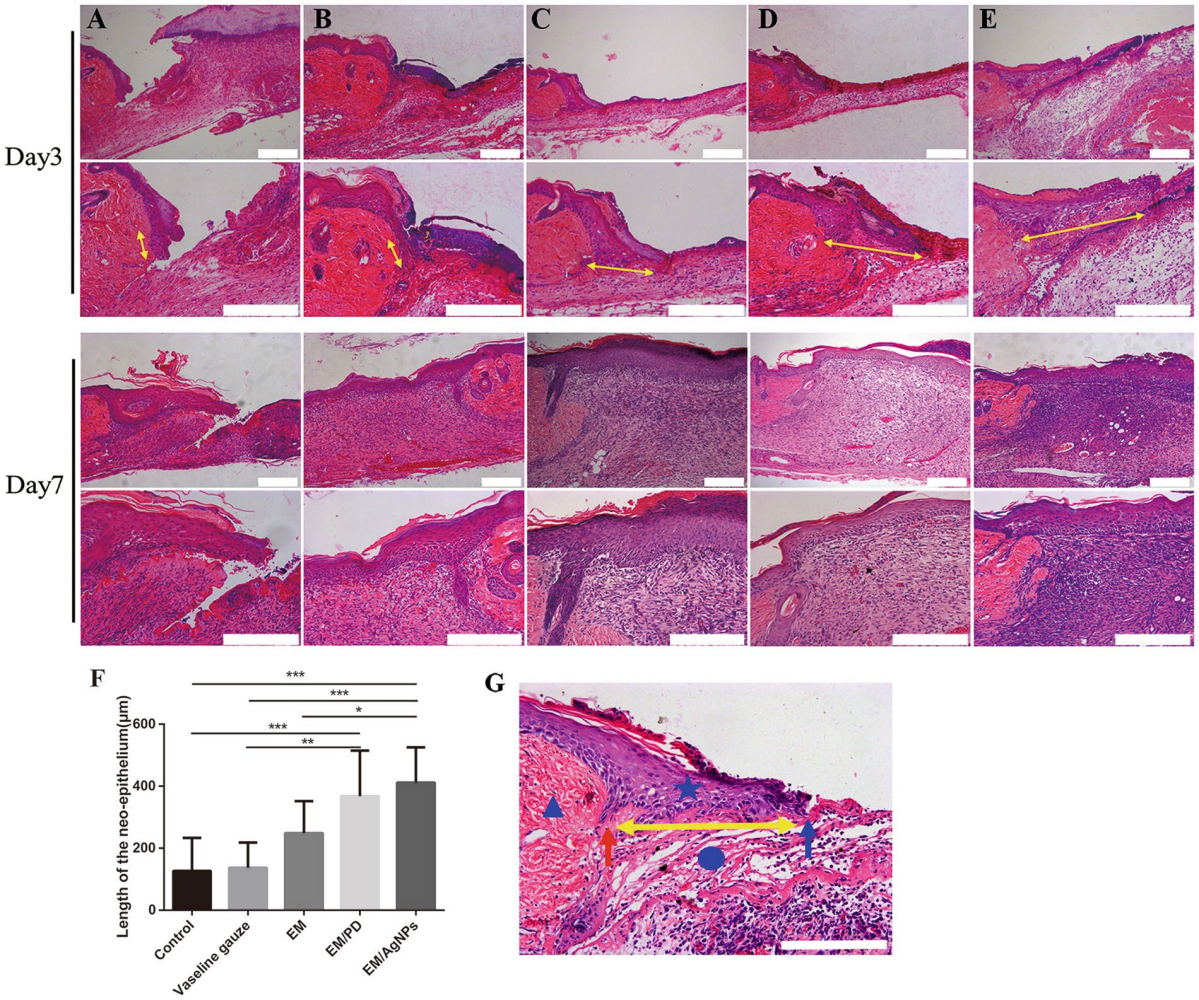
The effect of EM/AgNPs on re-epithelialization. Representative histological images (with H&E staining) of the length of the newly formed epidermis at days 3 and 7 post-surgery in the (**A**) control, (**B**) Vaseline gauze, (**C**) EM, (**D**) EM/PD and (**E**) EM/AgNPs groups. (**F**) Measurement of the length of the newly regenerated epidermis at day 3 post-surgery. (**G**) Schematic representation of the newly regenerated epidermis: the triangle represents the unwounded skin tissue, the circle represents the wound area, the pentagram represents the newly formed epidermis, the red arrow indicates border between unwounded skin tissue and wound area, the blue arrow indicates the advancing edge of newly-formed epidermis, and the yellow double-headed arrows indicate the length of the newly regenerated epidermis. The values are shown as the mean ± SD (n = 5). Scale bars: 200 μm.

**Figure 10:**
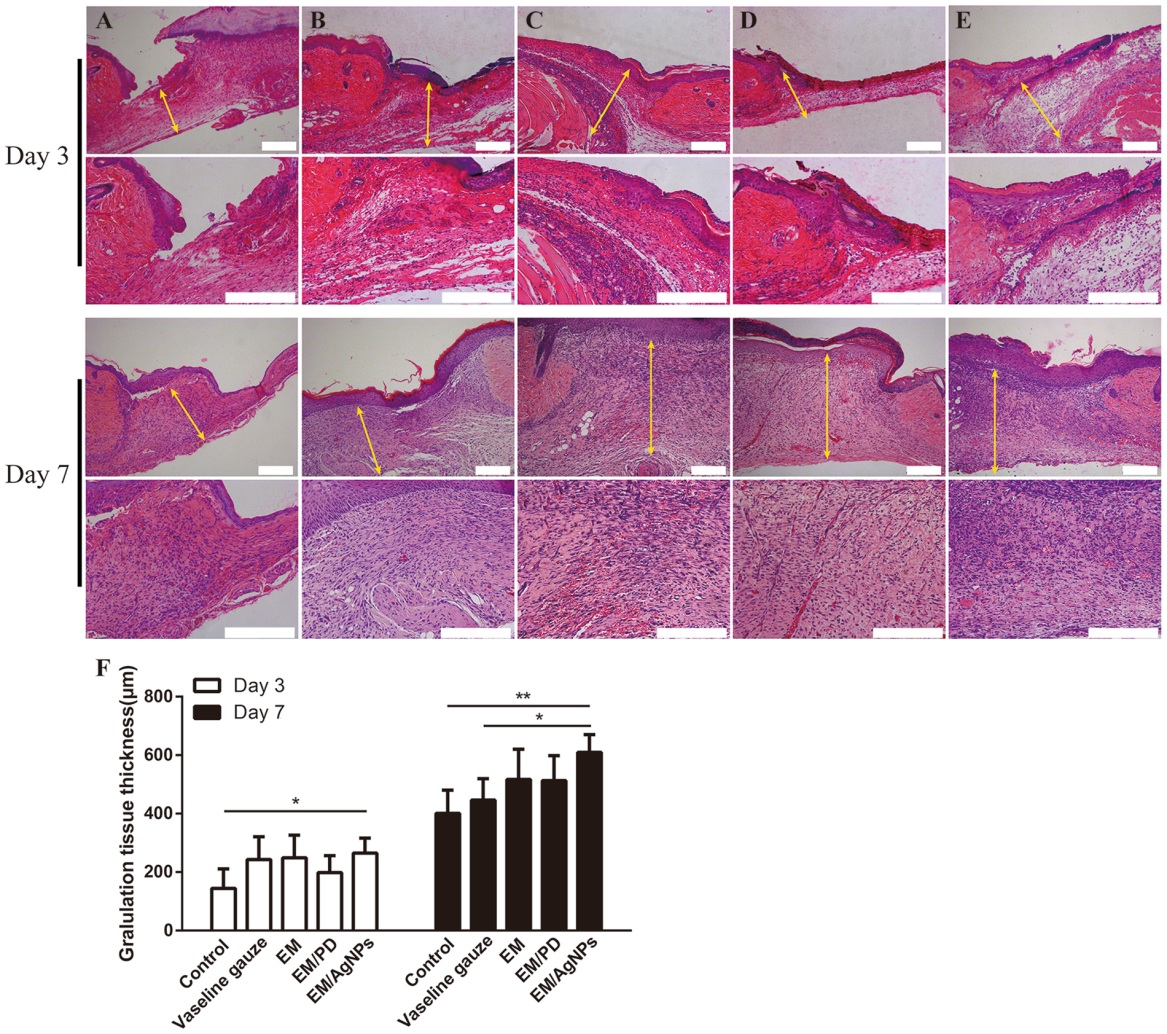
The effect of EM/AgNPs on granulation tissue formation. Representative histological images (with H&E staining) of the granulation tissue thickness at days 3 and 7 post-surgery in the (**A**) Control, (**B**) Vaseline gauze, (**C**) EM, (**D**) EM/PD and (**E**) EM/AgNPs groups. The yellow double-headed arrows indicate the granulation tissue. (**F**) Measurement of the granulation tissue thickness at days 3 and 7 post-surgery. The values are shown as the mean ± SD (n = 5). Scale bars: 200 μm.

As re-epithelialization and granulation tissue formation are necessary factors for wound healing, the length of newly regenerated epidermis and the thickness of granulation tissue were further histologically analyzed. Based on the H&E-stained sections, we found that the length of newly regenerated epidermis in the EM/AgNPs group was significantly longer than that in the control, Vaseline gauze and EM groups at day 3 post-surgery (Fig. 9). Additionally, EM/PD significantly promoted neo-epithelial regeneration compared with the control and Vaseline gauze groups (Fig. 9F). There was no significant difference in the length of the newly-regenerated epidermis between EM/AgNPs and EM/PD. On the other hand, EM/AgNPs obviously promoted granulation tissue formation at days 3 and 7 compared with the control group (Fig. 10). No significant differences were observed in granulation tissue thickness among the other groups (Fig. 10F).

### Effect of EM/AgNPs nanocomposite on cell proliferation and inflammation *in vivo*

#### Effect of EM/AgNPs nanocomposite on cell proliferation

**Figure 11:**
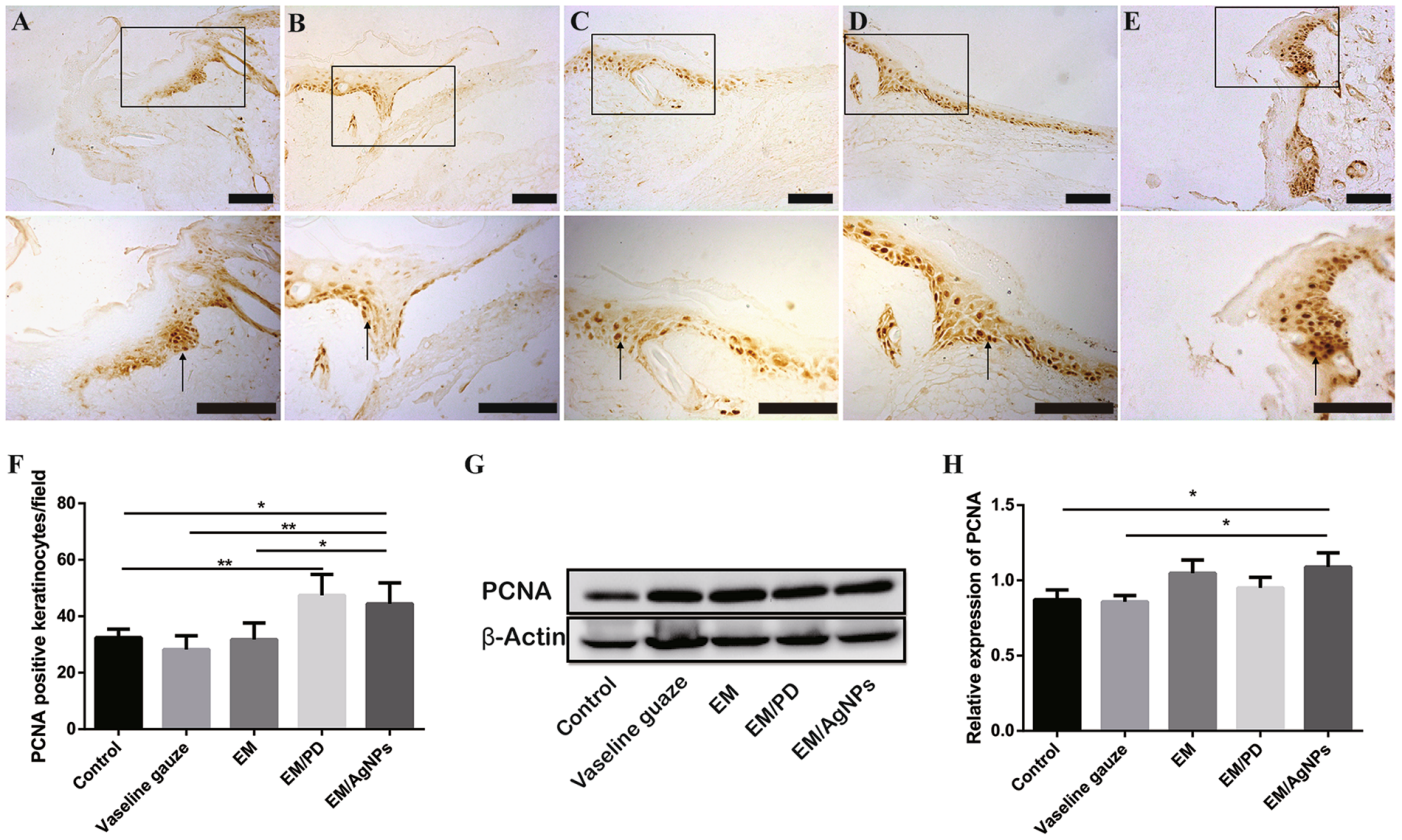
The detection of PCNA in wound tissue at day 3 post-surgery. Representative images of immunohistochemical staining of PCNA in the (**A**) Control, (**B**) Vaseline gauze, (**C**) EM, (**D**) EM/PD and (**E**) EM/AgNPs groups. The rectangular insets indicate magnified areas, and the black arrows indicate PCNA-positive keratinocytes. (**F**) The number of PCNA-positive keratinocytes per field in newly generated epidermis. The values are shown as the mean ± SD (n = 5). (**G**) The levels of PCNA protein in the full-thickness wound tissues, as detected by Western blotting, and (**H**) the optical density values of the PCNA bands. The values are shown as the mean ± SD (n = 3). Scale bars: 100 μm.

To further investigate the underlying mechanism of the observed effects, PCNA was selected as a cell proliferation marker and was detected by both immunohistochemistry and Western blotting at day 3 post-surgery^38^. As shown in the immunohistochemical images (Fig. 11A–F), significantly more positive keratinocytes were present at the wound edge in the EM/AgNPs and EM/PD groups than in the control, Vaseline gauze and EM groups. Furthermore, the Western blotting analysis showed that the level of PCNA protein isolated from full-thickness wound tissue was also significantly higher in the EM/AgNPs group than in the control group (Fig. 11G,H).

#### Effect of EM/AgNPs nanocomposite on inflammation

**Figure 12:**
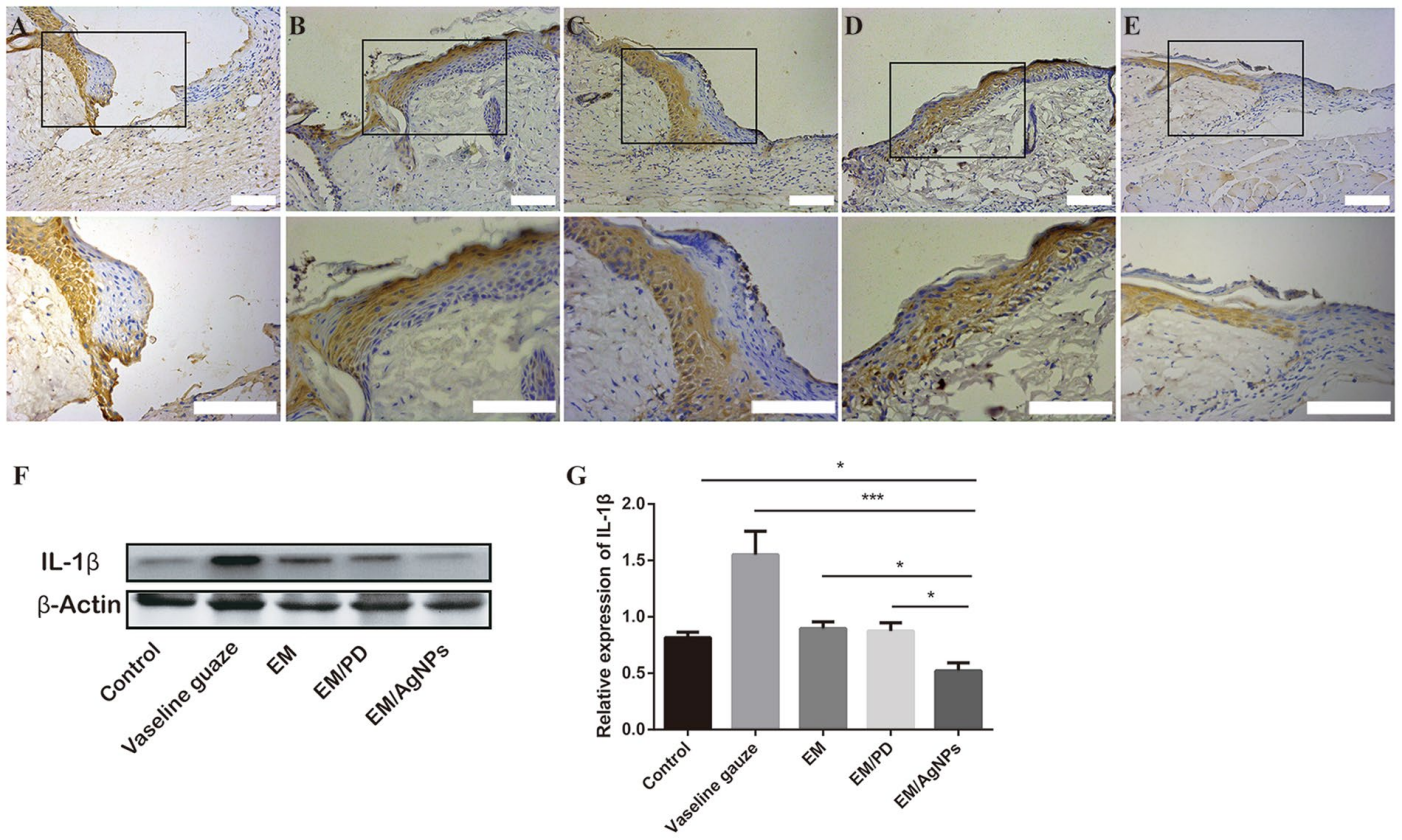
The detection of IL-1β in wound tissue at day 3 post-surgery. Representative images of immunohistochemical staining of IL-1β in the (**A**) Control, (**B**) Vaseline gauze, (**C**) EM, (**D**) EM/PD and (**E**) EM/AgNPs groups (n = 5). The rectangular insets indicate magnified areas. (**F**) The levels of IL-1β protein in the full-thickness wound tissues, as detected by Western blotting, and (**G**) the optical density values of the IL-1β bands. The values are shown as the mean ± SD (n = 3). Scale bars: 100 μm.

As IL-1β is an important indicator of inflammation, IL-1β levels were determined by immunohistochemistry and Western blotting at day 3 post-surgery^39^. As shown in Fig. 12A–E, IL-1β staining was strong in sections from the control, Vaseline gauze, EM and EM/PD groups, but it was weak in the EM/AgNPs group. Meanwhile, a quantitative analysis of the Western blotting results revealed that IL-1β expression was significantly lower in the EM/AgNPs group than in the other groups (Fig. 12F,G). Interestingly, we also observed that IL-1β expression was highest in the Vaseline gauze group. No significant differences in the IL-1β level were found among the control, EM and EM/PD groups (Fig. 12G).

## Discussion

Wound dressings containing AgNPs can effectively protect an injury from bacterial infection and promote tissue regeneration during the wound healing process^6, 17^. However, AgNPs are toxic to eukaryotes, and the cytotoxicity of such dressings should be taken into serious consideration^13^. Recent studies have found that the cytotoxicity of AgNPs is dose dependent and that low doses could be nontoxic^15, 16^. Thus, in the present study, we aimed to prepare a biocompatible antibacterial wound dressing by incorporating an optimized dose of AgNPs into a microfibrous EM network using mussel-inspired DA. Herein, the advantages of using DA to incorporate AgNPs are as follows: (1) DA can self-polymerize on various material surfaces, anchoring biomolecules or cells (Fig. 2H); (2) the reductive property of DA is effective enough for *in situ* AgNP formation (Fig. 3G); (3) DA is biocompatible and relatively nontoxic to the human body (Fig. 2J); and (4) the assembly process of AgNPs is simple and mild at room temperature^33, 40^.

To prepare the optimized EM/AgNPs, we first investigated the cytotoxicity of different AgNPs concentrations by observing cell attachment, and the dose of 30 μM was selected due to its optimal biocompatibility and effective antibacterial activity (Fig. 2A–H). The MTT assay further demonstrated that the resulting EM/AgNPs had no visible cytotoxic effects on fibroblasts (Fig. 2J). Our results were in line with those of previous reports showing that the cytotoxicity of AgNPs was dose dependent and observable only at high concentrations^15, 16^, indicating that the optimized EM/AgNPs were biocompatible. In addition, consistent with previous reports, we found that EM/PD could enhance cell attachment and viability compared with EM^41–44^. This is contributed to the adsorption and immobilization of serum proteins on PD coating, which leading to better cell adhesion, spreading and growth on the surface of EM/PD^43, 44^. Our result further indicates that application of mussel-inspired DA is an effective and safe strategy for tissue engineering.

Subsequently, the EM/AgNPs composite was characterized by SEM, STEM and FTIR. The SEM images showed the EM/AgNPs nanocomposite maintained the microfibrous network structure and appropriate porosity of EM after AgNPs deposition (Fig. 3C), which might be beneficial for the exchange of nutrients and metabolites, thereby promoting wound healing^5, 6^. The STEM images revealed the typical morphology of the AgNPs (Fig. 3G), and the average AgNPs diameter (13.9 nm) was in agreement with the reported size of AgNPs (less than 100 nm)^13^. The EDS analysis confirmed that AgNPs were successfully immobilized on the EM surface (Fig. 3I). Furthermore, the FTIR analysis revealed that the surface structures of EM/PD and EM/AgNPs were different, which also confirmed the coating of PD and immobilization of AgNPs (Fig. 4). Together, the SEM, STEM and FTIR results indicate that we successfully fabricated an EM/AgNPs nanocomposite for wound dressings via an *in situ* method.

Sustained and steady silver release is crucial for a wound dressing with a prolonged antibacterial role as well as for controlling the silver release in a safe and efficient manner. Figure 5B shows the burst release of silver from EM/AgNPs at day 1 (0.35 μg/mL), indicating that EM/AgNPs can efficiently and quickly prevent bacterial invasion when covering a wound site because silver ions can inhibit bacterial growth at concentrations were above 0.1 ppb^45^ (1 μg/mL = 1 ppm = 1000 ppb). Furthermore, constant silver ion release could still be observed at day 7, suggesting that EM/AgNPs had prolonged and steady antibacterial activity, which could continually protect cutaneous wounds from infection. More importantly, the concentration of released silver in our study is well below the previously reported toxic concentration (10 μg/mL)^46, 47^, indicating that the prepared EM/AgNPs can be applied without harmful effects. Therefore, this EM/AgNPs composite is a promising candidate for wound dressings that efficiently and continually prevent bacterial infection in humans.

The actual antibacterial activity of EM/AgNPs was detected by disc diffusion and bacterial suspension assays. Both methods revealed that EM/AgNPs were effective activity against *E. coli* (gram-negative bacteria) and *S. aureus* (gram-positive bacteria) (Figs 6 and 7). Our results indicate that the antibacterial activity of EM/AgNPs is strong enough to protect skin wounds from bacterial infection, which may efficiently enhance wound healing. Interestingly, consistent with previous studies, we also observed that EM/AgNPs showed better antibacterial activity against *E. coli* than *S. aureus*, which may due to the different cellular wall structures of gram-negative and gram-positive bacteria^7, 48^.

After evaluating the biocompatibility and antibacterial activity of EM/AgNPs, the effect of EM/AgNPs on wound healing *in vivo* was investigated using a murine full-thickness skin defect wound model. The results showed that EM/AgNPs could significantly promote wound healing compared with the control, Vaseline gauze, EM and EM/PD (Fig. 8). The histological analysis also revealed that the length of newly regenerated epidermis and the thickness of granulation tissue, two important parameters for wound healing, were significantly increased in the EM/AgNPs group compared with control group (Figs 9 and 10). Therefore, EM/AgNPs can provide effective support for re-epithelialization and granulation tissue formation to accelerate wound healing, and these benefits are likely related to the effective antibacterial activity and good biocompatibility of the EM/AgNPs composite^6, 49^.

To further investigate the underlying mechanism of the observed effects, the protein level of PCNA, a cell proliferation marker, was detected by immunohistochemistry and Western blotting. Consistently, EM/AgNPs could promote PCNA expression both in newly regenerated epidermis and in full-thickness wound tissue (Fig. 11), suggesting that the enhanced re-epithelialization and granulation tissue formation may result from promoted cell proliferation *in vivo* via treatment with EM/AgNPs. We also found that EM/PD promoted new epithelium formation, which was in agreement with the enhanced cell proliferation observed with EM/PD *in vitro* (Fig. 2J). We speculate that the better cytocompatibility and adhesive property of EM/PD than EM might be responsible for the better wound healing *in vivo* under EM/PD dressing than that under EM dressing^50^. It should be noted that EM/AgNPs did not promote cell proliferation *in vitro* (Fig. 2J). Percival *et al*.^51^ reported that once the skin barrier was no longer intact, bacterial contamination could occur within seconds, which could potentially interfere with the normal wound healing process. As described above, EM/AgNPs exhibited effective antibacterial activity and good biocompatibility (Figs 2, 6 and 7) and could thus efficiently protect skin wounds from further bacterial infection without causing tissue toxicity. In this way, this composite could provide a relatively aseptic and natural microenvironment for tissue regeneration.

Wound healing can be generally divided into three distinct but overlapping stages: inflammation, cell and tissue proliferation, and remodeling. These stages proceed with complicated interactions and influence the wound healing process together^52^. Thus, after demonstrating that EM/AgNPs can promote cell proliferation *in vivo*, we assessed the level of IL-1β, an important indicator of inflammation, by immunohistochemistry and Western blotting. We found that EM/AgNPs could significantly decrease IL-1β production in wound tissue (Fig. 12). Previous reports showed that AgNPs were capable of inhibiting the inflammatory reaction to promote wound healing^53, 54^. Therefore, we speculated that EM/AgNPs could also promote wound healing via controlling the inflammation reaction *in vivo*. We found that level of IL-1β was much higher in the Vaseline gauze group than that in the control group, this may be due to the Vaseline gauze-induced inflammation or foreign-body reaction^55, 56^.

Rapid re-epithelialization and low inflammation response during the wound healing process are believed to be correlated with reduced hypertrophic scar formation after tissue repair^52, 57^. Since EM/AgNPs can significantly promote re-epithelialization and reduce the inflammation reaction, it is reasonable to postulate that EM/AgNPs might also inhibit scar formation to some extent, which merits further study.

## Conclusions

In this study, we prepared a mussel-inspired EM integrated with an optimized quantity of AgNPs. The environmentally friendly EM/AgNPs nanocomposite not only exhibited good biocompatibility, effective antibacterial activity, and anti-inflammatory properties but also could efficiently accelerate wound healing. EM/AgNPs can potentially be used as an ideal wound dressing for cutaneous wounds.

## Materials and Methods

### Materials and animals

Dopamine hydrochloride was purchased from Solarbio Science & Technology Co., Ltd. (Beijing, China), and silver nitrate (AgNO_3_) was purchased from Sangon (Shanghai, China). Fresh eggs were purchased from a local supermarket (Chongqing, China).

BALB/c mice (male, 20–25 g) and green fluorescent protein (GFP) transgenic neonatal mice were obtained from the Experimental Animal Department of the Third Military Medical University. All the animal experiments were approved by the Institutional Animal Care and Use Committee of the Third Military Medical University. The animals were raised individually in plastic cages for one week before the experiments and were fed with free access to both water and autoclaved standard rodent chow under standardized conditions (room temperature: 25 °C; relative humidity: 50%; and circadian rhythm: 12 h). All methods were performed in accordance with the guidelines of The Third Military Medical University.

### Preparation and characterization of the EM/AgNPs nanocomposite

#### Preparation of EM

Fresh eggshells were cleaned with Milli-Q water (18.2 mΩ-cm), and then the inner membranes were carefully removed from the eggshells using forceps. The obtained EMs were washed with DDH_2_O 3 times, cut into small pieces (10 × 10 mm), and then immersed into DDH_2_O or an aqueous solution of a pH value of 3 or 11 for 72 hours to dissolve residual calcium carbonate compounds and collagen; these samples were then observed by scanning electron microscopy (SEM).

#### Preparation of the EM/AgNPs nanocomposite

After immersion in a pH 3 aqueous solution for 72 h, EM specimens were harvested, washed 3 times with DDH_2_O, and were then immersed into a DA solution (2 mg/mL in 10 mM Tris-HCL, pH = 8.5) at room temperature for 16 h. Finally, the DA-treated membranes were incubated with an aqueous silver nitrate solution (AgNO_3_) at different concentrations (50 mM, 10 mM, 1 mM, 100 μM, 50 μM, 30 μM, 10 μM) for another 18 hours in darkness at room temperature. Then, the films with immobilized silver were washed with DDH_2_O three times to remove the residual silver ions and dried at room temperature. After the DA and AgNO_3_ treatments, the specimens were designated as EM/PD and EM/AgNPs and are referred to as such in the following discussion.

#### SEM observation

The morphological features of the EM, EM/PD and EM/AgNPs were characterized using SEM (Hitachi, S-3400N, Japan). Briefly, the specimens were dried and sputter-coated with Au for 180 seconds and observed under vacuum conditions by SEM.

#### Porosity measurement

The network porosity of the EM, EM/PD and EM/AgNPs was measured via image analysis using the following formula, as previously described^58^:

$$P=1-n/N$$

where P represents porosity, n represents the number of pixels corresponding to microfibers, and N represents the total number of pixels in the image.

#### Scanning transmission electron microscopy (STEM) observation

A scanning transmission electron microscope (Zeiss LIBRA 200 FEG, 200 kV) was used to detect the detailed morphology and size distribution of the AgNPs, and energy-dispersive X-ray spectroscopy (EDS) was used for a compositional analysis. Briefly, after the EM/AgNPs films were ultrasonicated in DDH_2_O, a drop of the prepared solution was deposited on a carbon-coated copper grid and dried at room temperature prior to the STEM analysis.

#### Fourier transform infrared (FTIR) spectroscopy

The FTIR spectra of the EM, EM/PD, and EM/AgNPs films were identified using a PerkinElmer FTIR spectrometer (100S).

#### Ag+ release test

To investigate the release of silver ions from the EM/AgNPs films, specimens (n = 3) were each cut into (10 × 10 mm) pieces and immersed in 6 mL of phosphate-buffered solution (PBS) at 37 °C in darkness. The total supernatant of each group was harvested at each determined points, i.e. day 1, 3, 5 and 7 after EM/AgNPs membrane immersed in PBS, and analyzed using inductively coupled plasma-atomic emission spectrometry (ICP-AES, Leeman, USA).

### Antibacterial activity of the EM/AgNPs nanocomposite

#### Disc diffusion method

To investigate the antibacterial activity of the EM/AgNPs nanocomposite, *Staphylococcus aureus* (*S. aureus*, ATCC 25923) and *Escherichia coli* (*E. coli*, ATCC 25922) were chosen as model gram-positive and gram-negative bacteria, respectively. Briefly, bacterial suspensions were standardized to 0.5 McFarland standards (10^8^ CFU/mL) and uniformly coated on Mueller-Hinton agar plates using sterile cotton swabs. The films were punched into 6-mm-diameter discs and immersed in 75% alcohol for 30 minutes for sterilization. Then, after being washed with PBS three times, the films were carefully placed on the top of the plates, and zone of inhibition (ZOI) was measured after 24 hours of incubation at 37 °C. Sterilized filter discs served as the negative control group, and EM and EM/PD films were also used as control groups.

#### Bacterial suspension assay

As reported by Reithofer *et al*.^11^, 200 μL of bacterial stock was mixed with 5 mL of Luriae-Bertani (LB) medium and shaken at 200 rpm at 37 °C overnight. Then, the LB bacterial suspension was diluted to the predetermined starting concentration (optical density at 600 nm; OD_600_ = 0.07), and 500 μL of bacterial solution was added into each well of a 24-well plate. Subsequently, a sterilized film 10 × 10 mm in size was immersed into the bacterial solution, and the plate was incubated in a shaker incubator at 50 rpm and 37 °C. After 24 h, 100 μL of bacterial solution was transferred into a 96-well plate, and the OD_600_ value was measured. Here, untreated bacterial solution served as the negative control group, and bacterial solutions treated with penicillin (1000 U/mL) or streptomycin (1000 μg/mL) served as positive control groups for *S. aureus* or *E. coli*, respectively.

### Cytotoxicity test

#### Cell culture

Fibroblasts were isolated from GFP transgenic neonatal mice as previously described by Cheng *et al*.^59^. First, skin tissue obtained from neonatal mice was washed with PBS three times and then immersed in 0.5 mg/mL dispase II (Sigma, USA) at 4 °C overnight to separate the epidermis and dermis. Second, after the epidermis was removed, the dermis was minced and digested in 1 mL of 0.25 mg/mL trypsin (Boster, China) for 10 min. Then, 3 mL of Dulbecco’s Modified Eagle’s Medium (DMEM, Gibco, USA) containing 10% fetal bovine serum (Gibco, USA) was added to discontinue the digestion, and the mixture solution was centrifuged at 1000 rpm for 6 min. Finally, the cells were collected and incubated in DMEM containing 10% fetal bovine serum, penicillin (100 U/mL) and streptomycin (100 μg/mL) in a 5% CO_2_ incubator at 37 °C.

#### Attachment observation

Third-passage fibroblasts were used for this assay. As described above, the films were punched into 6-mm-diameter discs and sterilized by 75% alcohol. After being washed with PBS three times, films were placed into the bottom of each well of a 96-well plate. Then 8 × 10^3^ cells were seeded on each film, and the plate was incubated at 37 °C in a 5% CO_2_ incubator. After three days, the morphology of the fibroblasts was observed by fluorescence microscopy (Olympus, Japan).

#### MTT assay

An MTT assay was used to further investigate the viability of fibroblasts cultured on the films. At day 3, the films were placed into a new 96-well plate containing 100 μL of DMEM in each well. Then, 10 μL of MTT solution (Vazyme, USA) was added to each well, and the plate was incubated at 37 °C for 4 h. Subsequently, the medium was removed, 100 μL of DMSO was added to each well, and then the plate was placed on a shaker at room temperature for 10 min to dissolve the reaction product. The absorbance was measured at 570 nm using an enzyme-linked immunosorbent assay reader (Thermo Varioskan Flash, USA).

### *In vivo* wound healing experiment

To detect the effect of EM/AgNPs on wound healing, a murine full-thickness skin defect wound model was used^60^. After BALB/c mice were anaesthetized with 1% pentobarbital via intraperitoneal injection, the dorsal surface was shaved and cleaned with 75% alcohol. Then, two 4-mm-diameter full-thickness wounds were prepared on either side of the back using a punch. Immediately, a 4-mm-diameter standard disc was placed close to the wound, which was then imaged using a digital camera to represent the starting wound area. The wounds were covered with Vaseline gauze, EM, EM/PD or EM/AgNPs, and then a piece of adhesive biological membrane (NPWT-1, Negative Pressure Wound Therapy Kit, China) was glued onto the surface of the films. Wounds covered only with an adhesive biological membrane served as controls. At days 1, 3, 5 and 7 post-surgery, the wounds were imaged, and the remaining wound areas were carefully measured using IPP 6.0 software, as previously described. All measurements using software were performed by two independent researchers. The wound closure area was calculated by the following formula:

$$ \% \,{\rm{of}}\,{\rm{closed}}\,{\rm{wound}}\,{\rm{area}}=(I-R)/I\times 100 \% $$

where I represents the number of pixels of the initial wound area, and R represents the number of pixels of the remaining wound area at the determined time.

### Hematoxylin-eosin (H&E) staining

After mice were sacrificed at days 3 and 7 post-surgery, the wound tissues were carefully harvested, fixed with 4% paraformaldehyde, embedded in paraffin, sliced at a thickness of 5 μm, and then stained with H&E. The length of the neo-epithelium and the granulation tissue thickness stained with H&E were measured using Image J software. Since several wounds exhibited complete re-epithelialization at day 7 post-surgery, the length of neo-epithelium was histologically measured only at day 3 post-surgery. The length of newly formed epidermis is defined as the distance from the border between unwounded skin tissue and wound area to the advancing edges of the epidermis^60^. All measurements using software were performed by two independent researchers.

### Immunohistochemistry

To detect cell proliferation and inflammation levels in wound tissue, proliferating cell nuclear antigen (PCNA) and interleukin (IL)-1β were stained by immunohistochemistry at day 3 post-surgery. Briefly, the sections were deparaffinized, rehydrated and then incubated in a 95 °C sodium citrate buffer bath for 15 min, followed by incubation with 3% H_2_O_2_ for 15 min in darkness at room temperature. After being washed with PBS three times, the sections were incubated in 10% normal goat serum (Zhongshan Bio-tech Co., Ltd., China) at 37 °C for 30 min and then incubated with primary antibodies (anti-PCNA antibody ab15497, 1:200 dilution, Abcam, UK; anti-IL-1β antibody ab28364, 1:800 dilution, Abcam, UK) at 4 °C overnight. The sections were washed with PBS three times and then incubated with biotinylated goat-anti-rabbit IgG antibody (Zhongshan Biology Company, China) at 37 °C for 30 min. After being washed with PBS again, the sections were incubated with avidin peroxidase reagent (Zhongshan Biology Company, China) at 37 °C for another 30 min. Finally, after being stained with a 3,3′-diaminobenzidine tetrahydrochloride solution, the sections incubated with an antibody against IL-1β and were counterstained with hematoxylin; all sections were imaged using an optical microscope (CTR6000, Leica, Germany). The number of PCNA-positive keratinocytes per field in the newly regenerated epidermis were counted and analyzed by two independent researchers.

### Western blotting

The levels of PCNA and IL-1β protein in the wound tissue were detected and quantified by Western blotting. Briefly, the full-thickness wound tissues at day 3 post-surgery were carefully biopsied and immediately placed in liquid nitrogen. After being ground into powder, the tissue was dissolved in 300 μL of pre-cooled RIPA lysis buffer (P0013B, Beyotime) containing phenylmethyl sulfonyl fluoride (ST506, Beyotime), and total protein was extracted. Then, the protein concentration was measured using a BCA protein test kit (P0010S, Beyotime). Subsequently, 50 μg of total protein was separated by 12% SDS-PAGE and transferred onto PVDF membranes (Millipore, USA). The membranes were immersed into 5% bovine serum albumin for 2 hours at room temperature and then incubated with primary antibodies (anti-PCNA antibody ab15497, 1:1000 dilution, Abcam, UK; anti- IL-1β antibody ab28364, 1:1000 dilution, Abcam, UK; anti-β-Actin antibody, 1:2000 dilution, Santa Cruz, USA) overnight at 4 °C. After incubation with goat anti-rabbit IgG secondary antibodies (1:5000, Boster, China) at room temperature for 1 hour, the membranes were washed with Tris-buffered saline containing 1% Tween five times. Then, the proteins on the membranes were detected using a chemiluminescent system (Pierce, USA). The Quantity One software was used to quantify the bands, and the protein levels of PCNA and IL-1β were normalized to that of β-Actin.

### Statistical analysis

All values are presented as the mean ± SD, and the results were analyzed by one-way ANOVA. P < 0.05 was considered statistically significant (*represents p < 0.05, **represents p < 0.01, and ***represents p < 0.001).
